# Phenotypic plasticity in opsin expression in a butterfly compound eye complements sex role reversal

**DOI:** 10.1186/1471-2148-12-232

**Published:** 2012-11-29

**Authors:** Andrew Everett, Xiaoling Tong, Adriana D Briscoe, Antónia Monteiro

**Affiliations:** 1 Department of Ecology and Evolutionary Biology, Yale University, 165 Prospect St, New Haven, CT 06511, USA; 2 Department of Ecology and Evolutionary Biology, University of California, Irvine, CA, 92697, USA

**Keywords:** *Bicyclus anynana*, Reaction norm, Ommatidia, Opsin, Sexual dimorphism, Temperature-size rule, Phenotypic plasticity, Body size, Allometry, Vision, Optics

## Abstract

**Background:**

Animals often display phenotypic plasticity in morphologies and behaviors that result in distinct adaptations to fluctuating seasonal environments. The butterfly *Bicyclus anynana* has two seasonal forms, wet and dry, that vary in wing ornament brightness and in the identity of the sex that performs the most courting and choosing. Rearing temperature is the cue for producing these alternative seasonal forms. We hypothesized that, barring any developmental constraints, vision should be enhanced in the choosy individuals but diminished in the non-choosy individuals due to physiological costs. As a proxy of visual performance we measured eye size, facet lens size, and sensitivity to light, e.g., the expression levels of all opsins, in males and females of both seasonal forms.

**Results:**

We found that *B. anynana* eyes displayed significant sexual dimorphism and phenotypic plasticity for both morphology and opsin expression levels, but not all results conformed to our prediction. Males had larger eyes than females across rearing temperatures, and increases in temperature produced larger eyes in both sexes, mostly via increases in facet number. Ommatidia were larger in the choosy dry season (DS) males and transcript levels for all three opsins were significantly lower in the less choosy DS females.

**Conclusions:**

Opsin level plasticity in females, and ommatidia size plasticity in males supported our visual plasticity hypothesis but males appear to maintain high visual function across both seasons. We discuss our results in the context of distinct sexual and natural selection pressures that may be facing each sex in the wild in each season.

## Background

Phenotypic plasticity is the ability of a single genotype to produce distinct phenotypes based on environmental cues experienced during development, such as temperature, diet, or exposure to sunlight [1]. Although not always adaptive, this ability exists in most organisms and is especially common in insects. The lifespan of insects often falls within the duration of distinct seasons in the year, and each season often requires different adaptations for survival and/or reproduction. When discrete morphs appear associated with each season the plasticity is called a seasonal polyphenism [2].

**Figure 1:**
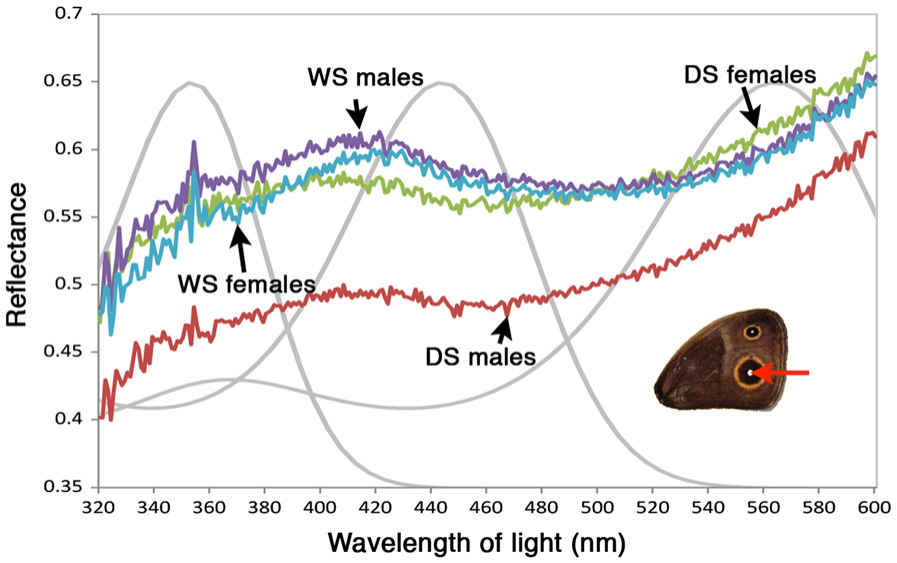
**Spectrophotometer measurements of average eyespot center brightness for each sex and seasonal form and hypothetical normalized absorbance spectra of *B. anynana *visual pigments.** Brightness measurements (average of 10 individuals per curve) correspond to the Cu1 dorsal forewing eyespot white centre. Details of the measurement can be found in supplemental materials and methods of [5]. Grey curves correspond to the absorbance spectra for the three visual pigments in *B. anynana*. Estimated λmax values for the long wavelength-absorbing pigment (560 nm) are from [6], and for the blue- (440 nm) and UV-absorbing pigments (350 nm), are based on intracellular recordings of other nymphalid butterflies [7,8].

In nature, the African butterfly *Bicyclus anynana* (Lepidoptera: Nymphalidae) has two distinct forms that are primarily cued by developmental rearing temperature predictive of Malawi’s two principal seasons—the dry (DS) and wet seasons (WS) [3]. The two seasonal forms differ in both morphology and behavior. In particular, the white, UV-reflective scales at the centre of the dorsal wing eyespots, the sexual ornaments [4], change in brightness across seasons and across sexes [5]. In particular, when brightness levels are integrated from 320 to 600 nm, the brightest eyespot centers are found in WS males, then in DS females, then in WS females, and finally in DS males (Figure 1). Plasticity in ornament brightness is associated with plasticity in sexual courtship roles and mate choosiness [5]. Males display their bright dorsal white spots and court slightly drabber, choosy females in the WS; whereas, females display the brighter ornaments and court much drabber, and choosy males in the DS [5] (Figure 1). This sex-role reversed species displays, thus, crossing reaction norms for each sex relative to courtship, choosiness, and ornament brightness, with changes in rearing temperature.

Given the sexual dimorphism and plasticity in sexual ornament brightness and mate choosiness, we posited whether similar crossing reaction norms could be detected in the visual system of these butterflies. In particular, we asked whether this butterfly might have evolved a temperature-sensitive, plastic mechanism for the development of its visual system in order to allow the choosy sex in each season to better evaluate the sexual ornament in potential mates, or allow the non-choosy sex to reduce expenditures on its visual system.

Phototransduction and vision entail significant metabolic costs [9,10]. Increasing overall eye size, and hence the number and/or size of an insect’s individual eye units, or ommatidia, is beneficial for vision [11-13], but larger fly photoreceptor cells (eight of these make up an ommatidia) are more costly to maintain both at rest and when active [14]. Because of these energetic costs, when the need for visual function is relaxed, eye size is greatly reduced or eliminated entirely, e.g. facet number decreases in *Drosophila* lines maintained in captivity [15], and parallel loss of eyes occurs in blind cave species [16].

Here we hypothesize that directional selection for enhanced vision in choosy *B. anynana* or relaxed selection [17] for vision in non-choosy *B. anynana* could be accompanied by plastic changes in eye size (via changes in the number or dimensions of the ommatidia) or in visual sensitivity (via changes in opsin levels). Visual sensitivity in vertebrate rods and cones is proportional to the product of the number of photoreceptor cells and the length of their outer segment [18,19]. So, changes in the number and length of the photoreceptor cells, perhaps by changes in overall eye size, may also result in changes in opsin expression, and thus, in visual sensitivity. Finally, the eyes of *B. anynana* express three opsin mRNAs encoding UV-, blue- and long-wavelength-absorbing (LW) visual pigments [6,20,21], and all three visual pigments can help detect the broad-spectrum UV-white light reflecting from the scales at the centre of the dorsal eyespots in *B. anynana* (Figure 1). So, in order to test for plasticity in eye size and/or visual sensitivity, we measured eyes, ommatidia number, individual facet lenses, and relative levels of UV, blue, and LW opsin mRNA molecules in adult males and females of the two seasonal forms.

## Methods

### Butterfly husbandry

Larvae were raised on young maize plants in two climate rooms, at 27°C and 17°C, respectively, with a 12:12 h light:dark cycle, and 80% relative humidity. Lighting was set to full at 6 am (with a gradual “sun-rise” starting at 5 am), and lighting was extinguished at 6 pm (with a gradual “sun-set” starting at 5 pm). Butterflies reared at temperatures above 24°C typically yielded a WS form, while those reared below 19°C yielded a DS form [22]. The adults were fed mashed banana and were sexed according to the presence or absence of androconia, which are scent organs on the wings of males.

### Eye size, wing size, facet lens area, and facet number measurements

In addition to measuring eye size and facet lens area in each animal, we also measured forewing area. Because *Bicyclus anynana* forewing area scales positively and strongly with body mass [23], we took this measurement as a proxy to examine how eye size scales with body size. All DS individuals used in the analysis were freshly frozen; whereas all WS individuals had been frozen for up to one year at −20°C prior to examination (Table 1). The latter butterflies showed no sign of any physical deformation after longer storage and were equally pliable upon thawing.

**Table 1:** Summary of specimen preparation and sample sizes used in this study

**Seasonal form/sex**	**Measurement type**	**Specimen preparation**	**Observations (N)**
Dry season females	Eye size	Freshly frozen	26
	Forewing size	Freshly frozen	28
	Facet size	Freshly frozen	10
	Opsin mRNA expression	Fresh, sacrificed on the 1^st^ day of emergence	3
Dry season males	Eye size	Freshly frozen	34
	Forewing size	Freshly frozen	34
	Facet size	Freshly frozen	19
	Opsin mRNA expression	Fresh, sacrificed on the 1^st^ day of emergence	3
Wet season females	Eye size	Frozen up to one year	26
	Forewing size	Frozen up to one year	26
	Facet size	Frozen up to one year	10
	Opsin mRNA expression	Fresh, sacrificed on the 1^st^ day of emergence	3
Wet season males	Eye size	Frozen up to one year	21
	Forewing size	Frozen up to one year	26
	Facet size	Frozen up to one year	10
	Opsin mRNA expression	Fresh, sacrificed on the 1^st^ day of emergence	3

**Figure 2:**
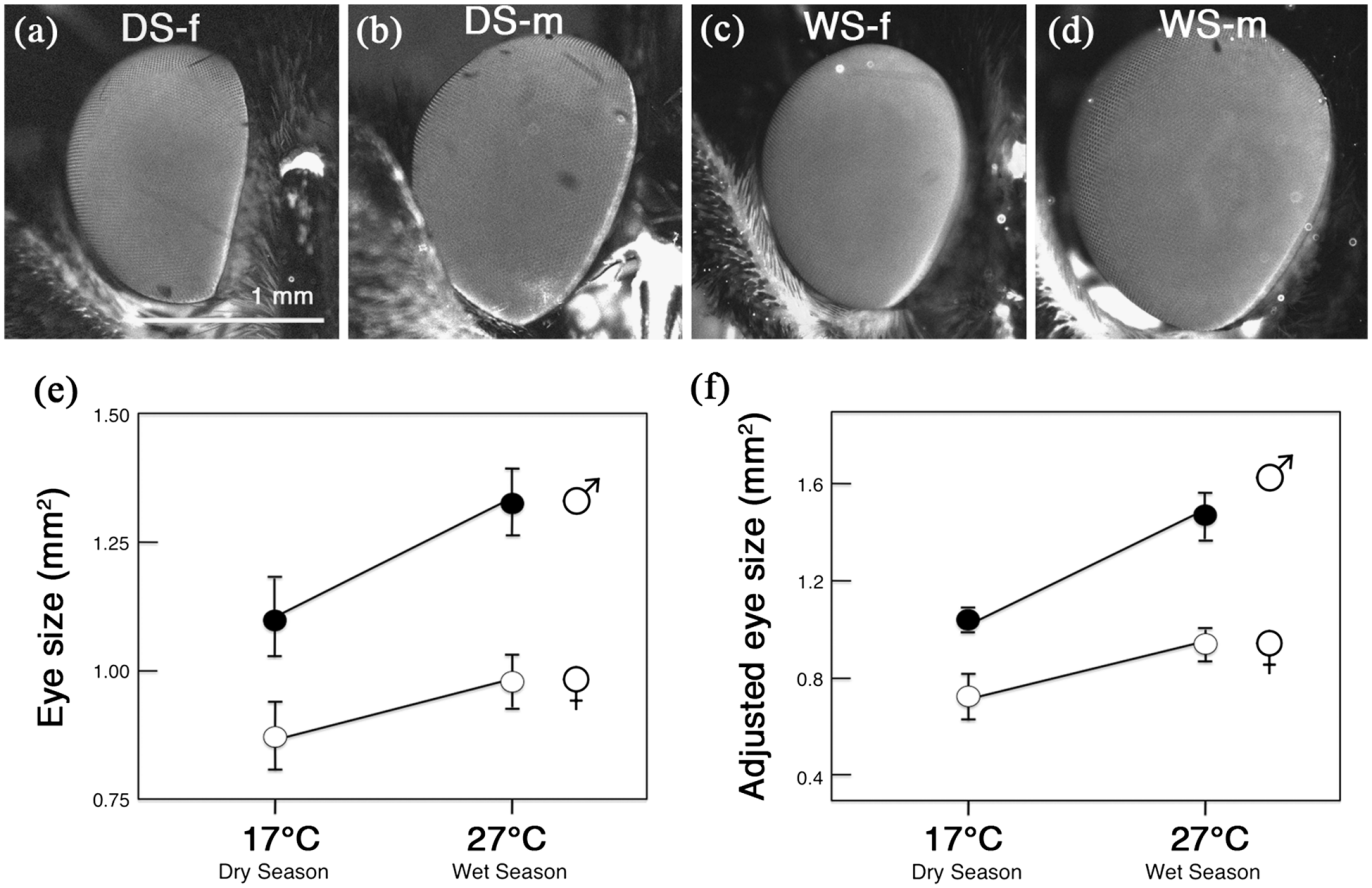
***B. anynana *is both phenotypically plastic and sexually dimorphic for eye size. (a)** Dry season female. **(b)** Dry season male. **(c)** Wet season female. **(d)** Wet season male. **(e)** Absolute mean eye size for each sex and seasonal form. **(f)** Relative mean eye size (corrected for body size) for each sex and seasonal form, using forewing area as a covariate, and evaluated at a wing area of 164.20 mm^2^. Error bars indicate 95% CI of means. Scale bar applies to all images in a-d.

To measure eye size, wing size, and facet lens area, individuals were placed under a Zeiss Discovery V8 SteREO scope and photographed with a Carl Zeiss AxioCam MRC camera (Figure 2a-d). To measure wing area, the right forewing was photographed under a 0.3X objective, using a 1X zoom, and a 10X eyepiece. To measure eye surface area, specimens were illuminated with blue light and photographed on their right side with a 1.5X objective, a 3.2X zoom, and a 10X eyepiece, through a green band-pass filter to make them stand out from the background. The two-dimensional pixel area of each eye or right forewing was quantified using Adobe Photoshop CS with the magic wand selection tool and later converted to μm^2^ or mm^2^ using a size standard.

To measure the surface area of an individual ommatidial facet, a new set of photos of the lateral-most surface of the eye, e.g. 0° latitude and +90° longitude [24,25], of each eye was taken at higher magnification under a 1.5X objective, using an 8X zoom, and a 10X eyepiece. Subsequently, in Photoshop, a box of known area (61,802 μm^2^) was used to enclose a group of facets, and the facets within that box were counted. Facets that were not fully surrounded by the box were given a count of 0.5 each. The individual facet surface area (in μm^2^) was then calculated by dividing the area of box by the number of facets within the box. Finally, facet lens area was converted to facet diameter using a circular approximation for facet shape. The approximate number of facets in each eye was calculated by dividing the total area of the eye (in μm^2^) by the facet lens area (in μm^2^). Our estimated total number of facets per eye is expected to deviate from the actual number because: 1) eye surface area was measured using a 2-D image, which neglects spherical effects; and 2) facet size varies slightly across eye regions [24-26].

### Extraction of total RNA, cDNA synthesis, and quantitative PCR

We collected the heads of three butterflies for each of the four butterfly groups as biological replicates: males zand females of DS and WS forms (total N=12). All twelve samples of mRNA were collected from freshly eclosed adults on the same morning, between 8 and 10 am, and heads were sampled at 10 am (± 10 min). The whole head of each butterfly was removed with a razor blade and immediately placed into a 1.5 ml RNAse free tube with lysis buffer RLT + β-mercaptoethanol (Qiagen, Valencia, CA). Tissue was lysed and mRNA extracted using the RNeasy Plus Micro kit (Qiagen, Valencia, CA). 2 μg of mRNA from each sample was then converted to cDNA using the High Capacity cDNA Reverse Transcription kit. cDNA was stored at −20°C at an approximate concentration of 100 ng/μl.

cDNA samples were run on two MicroAmp optical 96-well reaction plates, each using TaqMan primers and probes specific to the *B. anynana* UV, blue, or LW opsin sequences previously deposited in Genbank [6], and to a highly conserved housekeeping gene, eukaryotic 18S ribosomal RNA (rRNA) gene (Table 2). The 18S rRNA primers were previously shown to recognize *Drosophila* and *B. anynana* 18S rRNA genes, and 18S appears to have a constant expression level across multiple distinct samples, serving as an adequate endogenous control [27]. We included two technical replicates per biological replicate to control for pipetting error. Each well contained sample cDNA, sense and antisense primers, probes, and the TaqMan Universal PCR Master Mix according to the Gene Expression Assays protocol (Applied Biosystems, Foster City, CA). Plates were sealed with MicroAmp optical adhesive film (Applied Biosystems, Foster City, CA). The reaction was run for forty cycles according to the manufacturer's directions on an ABI 7500 Fast Real-Time PCR System, and results were analyzed with Sequence Detection System software (Applied Biosystems, Foster City, CA). Relative quantification of UV, blue, and LW opsin transcripts was obtained using the 2^-ΔΔCT^ method [28] in which expression levels were normalized first against 18S rRNA levels, and then against the normalized opsin levels of a randomly picked sample (one of the dry season males).

**Table 2:** Primers and Taqman probes used in this study

**Gene**	**Primers**	**Taqman probe**	**GenBank accession ID**
UV opsin	Forward primer: 5′-GCAAGCGAAGAAAATGAACGTAGAA-3′	5′-CTGCCGCGTTTTGAT-3′	AF484248.1
	Reverse Primer: 5′-CTATCCTGATTTCCGCTGACTCT-3′		
blue opsin	Forward primer: 5′-CGCGAGTGCAAGCATCTC-3′	5′-TTGCCGTTCACCTTCC-3′	AY918894.1
	Reverse primer: 5′-CACGAATTTTCCCCAGATCCTGAA-3′		
LW opsin	Forward primer: 5′-CGCCTGTGGAACCGATTACTT-3′	5′-TTGCCACGACTTGTCG-3′	AY918895.2
	Reverse primer: 5′-AGCAGAAGATCGAGTAGAACAGGAT-3′		

These sequences were used to quantitatively amplify *B. anynana* UV, blue, and LW opsin cDNAs. Primers and probes for eukaryotic 18S rRNA are proprietary (Applied Biosystems).

### Statistical analysis

Statistical analyses were performed on PASW18 and JMP 9 software packages. We tested for differences in absolute and relative eye size, lens surface area, total lens number, and relative quantification of opsin mRNA transcripts (2^-ΔΔCT^ values [28]) by performing a full factorial general linear model (GLM) analysis with sex and seasonal form as fixed factors. Changes in eye size relative to body size were tested in a full factorial GLM analysis of covariance, using forewing area as the covariate. PCR technical replicates for each biological sample were first averaged before being used in the GLM analysis.

## Results

### Eye size is sexually dimorphic and plastic

The eyes of males were larger than those of females (F_1,99_ = 70.67, p<0.001), and WS individuals, reared at high temperature, had larger eyes than DS individuals (F_1,99_ = 23.79, p<0.001). There was no interaction between seasonal form and sex (F_1,99_ =2.96, p=0.089) (Figure 2a-e).

Eye size, when compared across animals with the same body size (e.g. forewing area), was still different between seasonal forms and sexes. Males’ forewings were smaller than those of females (F_1,110_ =205.73, p<0.001), and DS individuals had larger forewings than WS individuals (F_1,110_ = 51.55, p<0.001), with no seasonal form by sex interaction (F_1,110_ = 0.38, p=0.538). When corrected for body size, males had larger eyes than females (F_1,99_ = 73.36, p<0.001) and WS individuals had larger eyes than DS individuals (F_1,99_ = 26.84, p<0.001), with no significant interaction between seasonal form and sex (F_1,99_ = 0.96, p=0.330) (Figure 2f).

**Figure 3:**
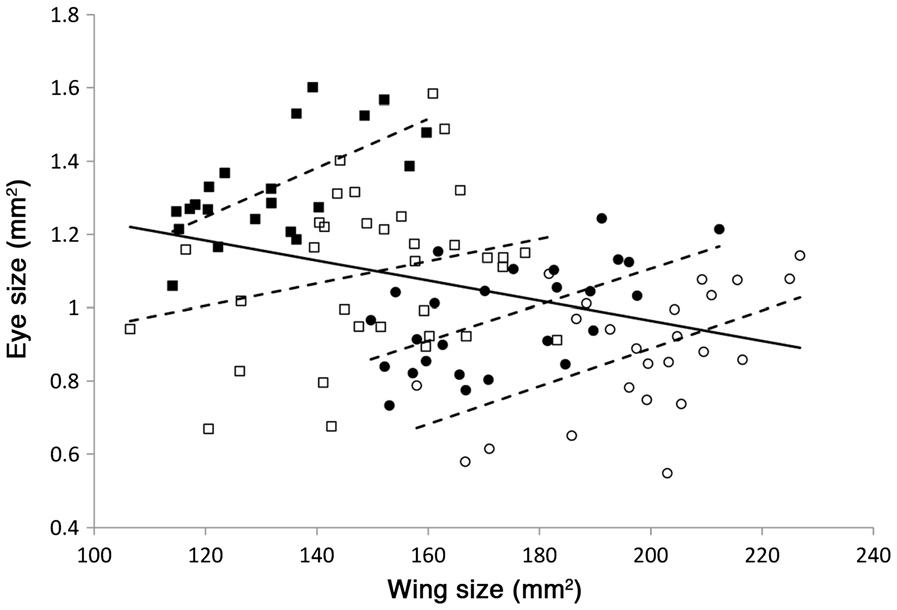
**Relationship between wing size and eye size across sexes and seasonal forms.** Eye size is positively correlated with wing size within a sex and a seasonal form (dashed lines). DS individuals, however, have larger wings and smaller eyes than their WS counterparts, leading to an overall negative correlation between wing size and eye size across all data points (black line) (males = squares; females = circles; open symbols = DS; closed symbols = WS).

Within each sex and seasonal form, individuals with larger wings had larger eyes (all significant correlations except for DS males: Pearson correlation for WS males = 0.658, p=0.001; WS females = 0.588, p=0.002; DS males = 0.253, p=0.149; DS females = 0.523, p=0.006) (Figure 3). However, when all points were analyzed together, wing size was negatively correlated with eye size (Pearson correlation = −0.354, p<0.001) (Figure 3). This means that eye size scales positively with body size within each temperature regime, but, as rearing temperature decreases, eyes become smaller as wings become larger. Males always have larger eyes and smaller wings than females regardless of temperature.

### Facet lens area and facet number are sexually dimorphic and plastic

**Figure 4:**
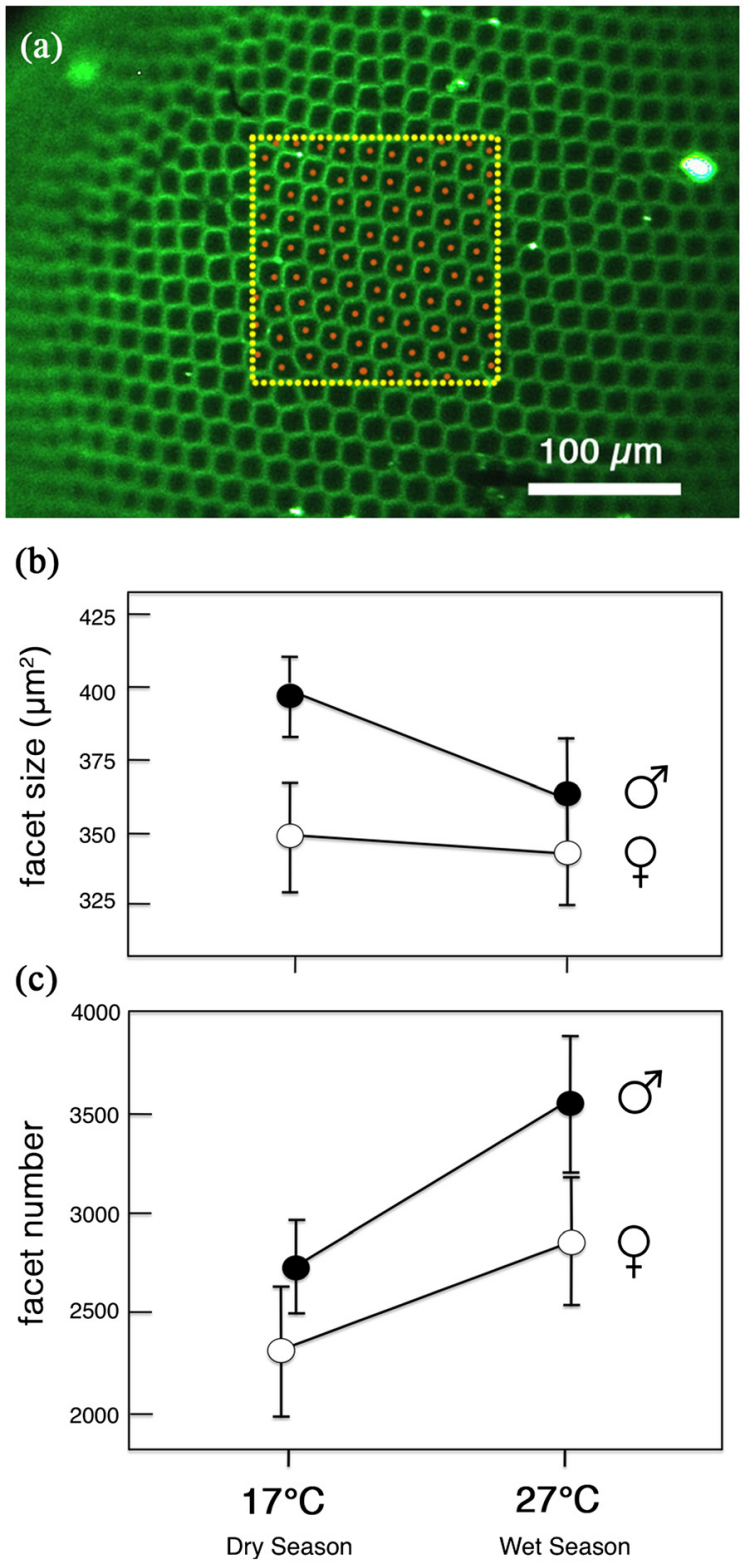
***B. anynana *is both phenotypically plastic and sexually dimorphic for facet lens area. (a)** Ommatidia of a wet season female specimen showing the lateral region of the eye in which facet lens area was calculated for each eye. **(b)** Mean facet size (lens area) for each sex and seasonal form. **(c)** Mean number of facets per individual per eye calculated from facet size and eye size. Error bars indicate 95% CI of means.

Facet lens area was both sexually dimorphic and plastic. Males had larger facets than females (F_1,44_ = 14.53, p < 0.001), and DS individuals (especially males) had larger facets than WS individuals (F_1,44_ = 5.00, p=0.031), with no interaction between sex and seasonal form (F_1,44_ = 2.50, p=0.121) (Figure 4b; Table 3). Facet size was not correlated with eye size both across all groups and within each sex and seasonal form (Pearson correlation for all data = 0.169, p=0.250; for WS males alone = −0.031, p=0.937; WS females = 0.063, p=0.862; DS males = −0.176, p=0.472; DS females = 0.223, p = 0.536). Males had a greater number of facets than females (F_1, 44_ = 13.06, p<0.001) and WS individuals had a greater number of facets than DS individuals (F_1,44,_ = 19.95, p<0.001) (Figure 4c).

**Table 3:** **Mean facet diameterof seasonal and sexual forms of *B. anynana***

**Seasonal form**	**Male**	**Female**
Dry season	22.464 (N=19)	21.044 (N=10)
Wet season	22.481 (N=9)	21.886 (N=10)

Measurements are in μm using a circular approximation for each facet. N=number of individuals measured. A range of 60–85 facets were counted per eye.

**Figure 5:**
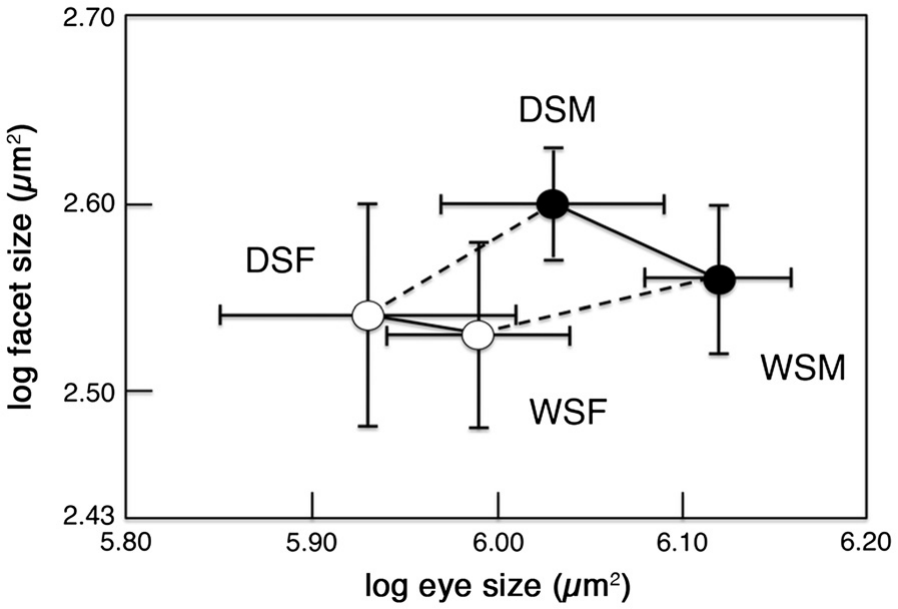
**Covariation of log-transformed body part size (eyes) and cell size (facets).** The solid lines represent the regressions between seasonal forms and the dotted lines represent the regressions between sexes. Black circles represent males and white circles represent females. The scale was adjusted so that a slope of 1.0 corresponds to a 45° angle. Error bars indicate 95% CI of means.

In order to better explore the relative contributions of facet size (a proxy for cell size) and facet number (a proxy for cell number) to eye size across rearing temperatures, we plotted the log of mean facet size by the log of mean eye size for each of our four samples (Figure 5). The slopes of the lines connecting these four points offers a quick visualization of how cell size versus cell number contributes to variation in eye size [29]. The two slopes connecting males and females within a seasonal form were positive (0.60, dry season; 0.23, wet season) and the two slopes connecting seasonal forms of the same sex were negative (−0.44, males; -0.17, females). This result indicates that, within a seasonal form, sexual differences in eye size were achieved both by changes in facet lens area and number. Within a sex, however, seasonal differences in eye size were achieved by changes in facet number, despite being counteracted by opposite changes in facet size, i.e., the larger wet-season eyes had more facets, which outweighed the effects of also having smaller facets.

### Opsin expression is sexually dimorphic and plastic

**Figure 6:**
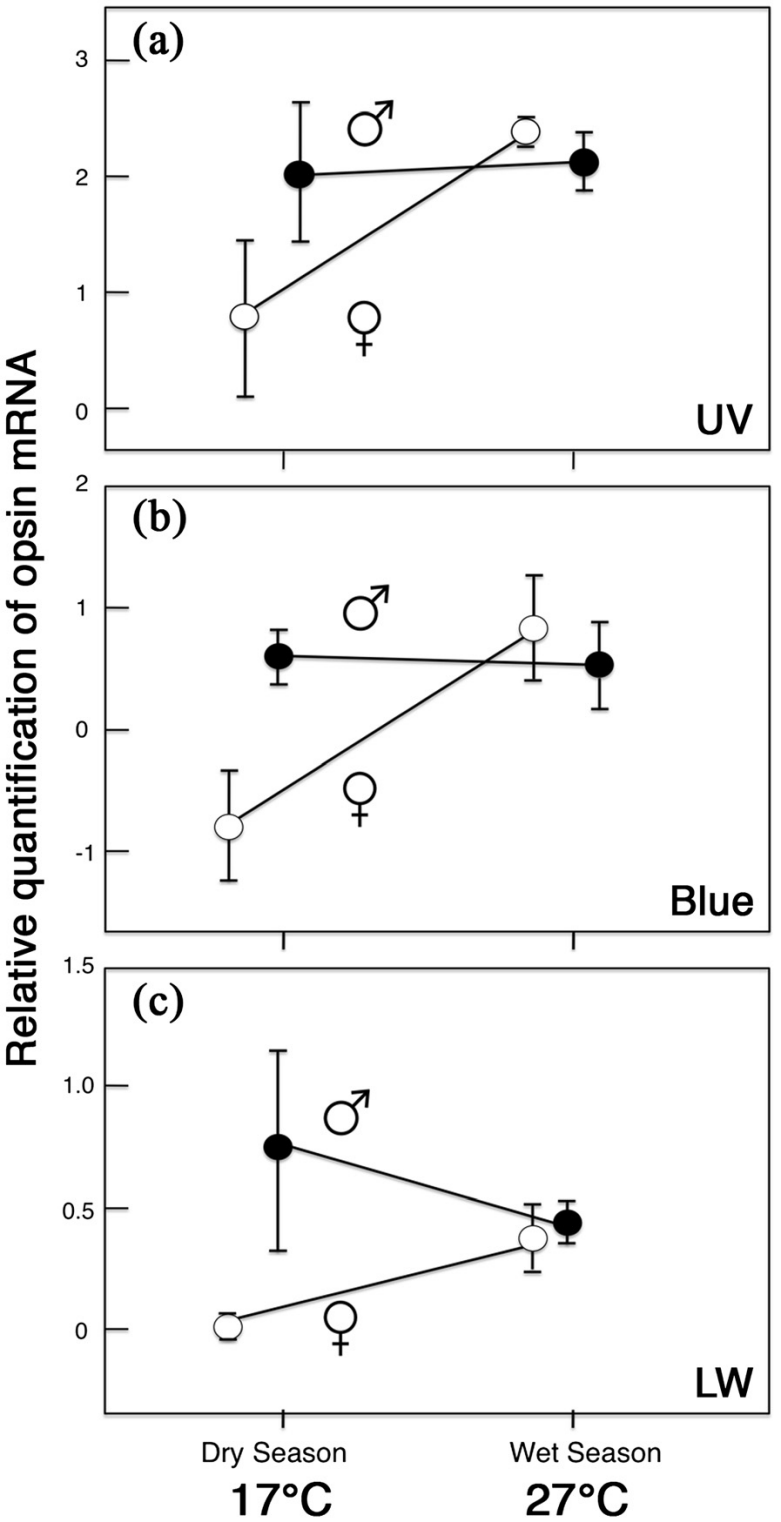
**Relative quantification of opsin mRNA levels in *B. anynana *males and females of the WS and DS forms. (a)** UV opsin mRNA expression. **(b)** Blue opsin mRNA expression. **(c)** LW opsin mRNA expression. Relative expression levels obtained using the 2^-ΔΔCT^ method. Scales cannot be compared between panels. Error bars indicate 95% CI of means produced from different biological samples.

*B. anynana* is sexually dimorphic and phenotypically plastic for log_10_-transformed opsin mRNA expression upon adult emergence from the pupal stage. The sexual dimorphism, however, is only present in the DS, and the plasticity is only present in females, due to a significant season by sex interaction (UV: F_1,8_=10.30, p=0.0124; blue: F_1,8_=19.87, p=0.0021; LW: F_1,8_=21.11, p=0.0008) (Figure 6). In the DS, males expressed higher UV, blue, and LW opsin levels than females (UV: F_1,5_=7.59, p = 0.051; blue: F_1,5_=28.94, p=0.006; LW: F_1,5_=31.60, p = 0.005); whereas, significant sexual differences were not found in WS individuals (UV: F_1,5_=4.16, p=0.111; blue: F_1,5_=1.21, p=0.333; LW: F_1,5_=0.69, p=0.454). While males maintained high opsin expression across temperatures (UV: F_1,5_=0.07 p=0.799; blue: F_1,5_=0.11, p=0.757; LW: F_1,5_=1.23, p=0.329), females transitioned from low expression in the DS to high expression in the WS (Figure 6) (UV: F_1,5_=21.83 p=0.010; blue: F_1,5_=26.40, p=0.007; LW: F_1,5_=27.05, p=0.007). In summary, lower rearing temperature led to lower levels of UV, blue, and LW opsin mRNA expression in a female’s but not in a male’s eye, creating sexual dimorphism in the DS, but not in the WS. This finding suggests that DS females are also producing lower amounts of these important visual pigment proteins relative to the other three groups; although, we did not measure opsin protein levels directly. For a summary of all results described in the multiple sections above, see Table 4.

**Table 4:** Summary of findings

**Experiment**	**Groups compared**^**†**^	**p-value**^**‡**^	**Pearson’s correlation coefficient (PCC)**	**PCC result or group with greater mean, if significant**
**Unadjusted eye size**	M vs. F	<0.001***		M
	WS vs. DS	<0.001***		WS
**Forewing area**	M vs. F	<0.001***		F
	WS vs. DS	<0.001***		DS
**Adjusted eye size (corrected with forewing area)**	M vs. F	<0.001***		M
	WS vs. DS	<0.001***		WS
**Correlation between eye size and wing size**	WSM	0.001**	*r*=0.658	Linear relationship
	WSF	0.002**	*r*=0.588	Linear relationship
	DSM	0.149	*r*=0.253	
	DSF	0.006**	*r*=0.523	Linear relationship
	Both sexes, both seasons	<0.001***	*r*=−0.354	Linear relationship
**Facet lens area**	M vs. F	<0.001***		M
	WS vs. DS	0.031*		DS
**Facet number**	WSM	0.937	*r*=−0.031	
	WSF	0.862	*r*=0.063	
	DSM	0.472	*r*=−0.176	
	DSF	0.536	*r*=0.223	
	Both sexes, both seasons	0.250	*r*=0.169	
	M vs. F	<0.001***		M
	WS vs. DS	<0.001***		WS
**UV opsin expression**	DSM vs. DSF	0.051		DSM (not significant)
	WSM vs. WSF	0.111		
	DSM vs. WSM	0.799		
	DSF vs. WSF	0.010*		WSF
**Blue opsin expression**	DSM vs. DSF	0.006**		DSM
	WSM vs. WSF	0.333		
	DSM vs. WSM	0.757		
	DSF vs. WSF	0.007**		WSF
**LW opsin expression**	DSM vs. DSF	0.005**		DSM
	WSM vs. WSF	0.454		
	DSM vs. WSM	0.329		
	DSF vs. WSF	0.007**		WSF

† M, male; F, female; DS, dry season; WS, wet season; DSM, dry season male; WSM, wet season male; etc. ‡ Not significant, p>0.05; *, p < 0.05; **, p < 0.01; ***, p < 0.001.

## Discussion

The eye is hailed as a paragon of organismal complexity, an organ of sophisticated design and many interacting parts [30]. Here we report how an insect’s eye morphology and physiology can be regulated by developmental rearing temperature in a fashion that is likely to be adaptive to adults emerging in alternating seasons.

Our work documents sexual dimorphism and plasticity in opsin levels in *B. anynana* in a complex way, cued by developmental rearing temperature. At low rearing temperature, males display significantly higher levels of Blue and LW opsin mRNA levels than females, and females reared at low temperature display significantly lower UV, Blue, and LW opsin mRNA levels than females reared at high temperature. We demonstrate, for the first time, that developmental rearing temperature induces changes in opsin expression levels in an insect’s eye. However, plasticity in type or level of opsin expression was previously documented in a variety of species responding to different environmental conditions: Opsin levels change with light rearing environment in African cichlids [31], with the diurnal cycle in *Limulus*[32], and with age in *Drosophila*[33]; whereas new opsins are induced upon maturation in the European eel [34], or upon a change in lifestyle in salmon [35]. In addition, sexual dimorphism in opsin spatial expression patterns [36] and in the presence/absence of a non-opsin filter pigment [37] were previously documented in butterflies.

According to our original hypothesis, and barring any developmental constraints, we expected *B. anynana* to have evolved plasticity in its visual system, due to physiological costs associated with vision [10]. In particular, we expected the visual system to decrease in capacity in non-choosy courters. We found that physiological and morphological changes conformed, in part, to our predictions.

The non-choosy DS females displayed lower levels of UV, blue, and LW opsin transcript than the choosy WS females. Assuming that opsin mRNA is being actively translated to protein, DS females appear to have reduced the costs associated with photoreceptor energy consumption [15] in exchange for reduced visual function. To measure the impact of opsin levels on visual function, *in vivo* intracellular microelectrode recordings would provide direct physiological information on the costs and benefits of opsin production in *Bicyclus*[14]. The DS females, who mate indiscriminately with males with or without the dorsal eyespot ornaments [5], can afford such loss of visual function. Males, however, including the equally indiscriminate WS males, maintained high levels of opsin expression across seasons and did not conform to our original predictions.

Our results for eye size and facet size plasticity also only partly support our original hypothesis. We predicted that choosy individuals should develop either higher acuity or greater sensitivity to light to evaluate the small dorsal eyespot centers, i.e. WS females and DS males should have more facets and/or larger facets than their non-choosy DS and WS same-sex forms. We found that facet size was especially large in the choosy DS males relative to WS males, and choosy WS females had more facets than non-choosy DS females, but the reverse prediction did not pan out for female facet size or male facet number. Facet number and facet size were always larger in males relative to females of both forms, and facet number was always larger in WS versus DS individuals, with no significant sex by season interaction. In order to explain these results, we sought alternative explanations for the observed patterns of sexual dimorphism and plasticity that move away from examining *B. anynana* eyes as useful only for evaluating sexual ornaments in the context of mate choice.

Smaller facets at warmer WS temperatures may be explained either by a biophysical constraint, the “temperature-size rule” [38], or by natural selection for improved light sensitivity in the DS or visual acuity in the WS, for both sexes. The “temperature size rule” states that the rate of cell division increases more than the rate of cell growth with increasing temperature, and helps explains the pervasive pattern of small bodies at high temperatures (but see [39] for a second explanation of this latter phenomenon). Animals experiencing high temperature during development, thus, will end up reaching maturity with the same number of cells but with smaller cells [40]. Natural selection-driven alternatives to this biophysical constraint, could be that larger facets, often associated with activity at lower light levels [41], are selected for in the DS, but currently we have no indication that light levels differ between the dry and wet seasons in Malawi, or that the two seasonal forms are active at different times of the day. Another natural selection-driven alternative is that smaller facets in the WS are actually adaptive as this leads to lower inter-ommatidial angles and improves visual acuity [42] in the WS.

Regardless of the forces driving smaller facet size in WS eyes, our study suggests that visual demands are lower in the DS because DS eyes are 13% smaller than WS eyes across sexes. Lower visual demands may be related to lower environmental complexity in the DS or with lower activity levels in DS butterflies. Lower temperature is known to decrease butterfly activity levels in the field [43,44], and lab observations of *B. anynana* have indicated that DS butterflies are generally less active than their WS counterparts. Larger eyes in the WS, on the other hand, would allow the more active, and thus more conspicuous, WS butterflies to search for mates and thwart predators more efficiently. Alternatively, visual demands in the DS are trading off against other energetically expensive demands, such as survival through the long dry season [3].

Sex-specific butterfly behaviors, beyond mate ornament discrimination, may also contribute to explain why the larger-bodied females have fewer facets than the smaller-bodied males, why opsin expression levels were high in males of both seasonal forms, and why DS females have the lower opsin levels of all four groups. Males of *B. anynana* had 28% larger eyes and 12% larger facets than the larger females across temperatures. This is a different pattern from that found in *Drosophila*, where the larger females also have larger eyes [45,46]. Our data, however, matches previous studies of eye size sexual dimorphism in other butterfly species [47-49], and, although developmental constraints cannot be ruled out, the dimorphism is likely to be the result of male-limited activities such as mate searching, and territory defense typical of satyrid butterflies [44,50]. These activities, as well as their side effects of becoming more visible to predators, may require males to maintain large eyes and high opsin expression across seasons. *B. anynana* males have a typical perch-and-chase strategy [51], and males with better vision are expected to have an advantage at detecting passing females, competing males, or nearby predators. The plastic courtship roles may take over only once males have localized a female [5]. On the other hand, female-limited searches for oviposition sites, which may be suspended in the DS (due to ovary dormancy [3]), may lead, through relaxed selection, to reductions in eye size and opsin levels in DS females due to their high maintenance costs [10].

Plasticity of eye size in *B. anynana* appears to be operating through the control of resource allocation between different body parts. Resource competition between imaginal discs in holometabolous insects reared at a constant temperature can give rise to size trade-offs in adult body parts, such as eyes and wings. This competition takes place during the pre-pupal and pupal stages because growth of the imaginal discs happens in a closed system once the larva has stopped feeding [52-55]. It appears that in *B. anynana*, high rearing temperature is cueing development to shift resource allocation away from wings and into eyes (Figure 3). Additional experimentation will be required to test whether these plastic patterns of allocation from wings to eyes are adaptive.

## Conclusions

The eyes of *B. anynana* change their opsin gene expression profiles and their morphological characteristics in response to developmental rearing temperature. The plastic response of both males and females to the alternate rearing temperatures is congruent with our original hypothesis of non-choosy individuals having relaxed selection [17] on costly visual function. Overall, visual function was found to be lowest in the non-choosy, non-patrolling, and non-egg-laying dry season females relative to the other three groups. However, biophysical constraints, as well as additional ecological and behavioral factors may also help account for the data. The plasticity and sexual dimorphism documented for the *B. anynana* compound eye provide additional, compelling evidence for the remarkable level of adaptation and integration of disparate traits (wing pattern, behavior, life history, physiology, visual system) in a species that has evolved in a seasonal environment and that experiences recurrent distinct selection pressures acting on these traits at different times of the year [3,5,56,57]. While the primary selective forces shaping eye physiology and morphology in *B. anynana* have to be further pursued with field and lab experiments, we propose that an intricate balance between sexual selection, natural selection, and developmental constraint are playing a role.

## Competing interests

The authors declare that they have no competing interests.

## Authors’ contributions

AE carried out all aspects of the study and drafted the manuscript. XT helped with the qPCR experiments. ADB participated in the design of the study and helped draft the manuscript. AM conceived the study, participated in its design and coordination, and helped to draft the manuscript. All authors read, commented, and approved the final manuscript.

## Authors’ information

AE takes an interest in the evolution of adaptive phenotypic plasticity. XT is interested in the molecular basis of Lepidopteran development. ADB studies the evolution of insect vision. AM is interested in morphological evolution and especially in the evolution and development of butterfly wing patterns.
